# Cognitive bias modification for interpretation with and without prior repetitive negative thinking to reduce worry and rumination in generalised anxiety disorder and depression: protocol for a multisession experimental study with an active control condition

**DOI:** 10.1136/bmjopen-2016-013404

**Published:** 2016-12-16

**Authors:** Charlotte Krahé, Andrew Mathews, Jessica Whyte, Colette R Hirsch

**Affiliations:** 1 Department of Psychology, Institute of Psychiatry, Psychology and Neuroscience, King's College London, London, UK; 2 Department of Psychology, University of California, Davis, California, USA

**Keywords:** cognitive bias modification, interpretation bias, depression, generalized anxiety disorder, worry, rumination

## Abstract

**Introduction:**

Worry and rumination are two forms of repetitive thinking characterised by their negative content and apparently uncontrollable nature. Although worry and rumination share common features and have been conceptualised as part of a transdiagnostic repetitive negative thinking (RNT) process, it remains unclear whether they share the same underlying cognitive mechanisms. This multisession experimental study investigates the tendency to make negative interpretations regarding ambiguous information as a cognitive mechanism underlying RNT. We compare multisession cognitive bias modification for interpretations (CBM-I) with an active control condition to examine whether repeatedly training positive interpretations reduces worry and rumination in individuals with generalised anxiety disorder or depression, respectively. Further, we examine the potential modulatory effects of engaging in RNT immediately prior to CBM-I.

**Design, methods and analysis:**

A community sample of individuals meeting diagnostic criteria for either generalised anxiety disorder (n=60) or current major depressive episode (n=60) will be randomly allocated to CBM-I with prior RNT, CBM-I without prior RNT (ie, standard CBM-I), or an active control (no resolution of ambiguity) condition. All conditions receive a 3-week internet-based intervention consisting of one initial session at the first study visit and nine home-based sessions of CBM-I training (or active control). We will assess and compare the effects of CBM-I with and without prior RNT on ‘near-transfer’ measures of interpretation bias closely related to the training as well as ‘far-transfer’ outcomes related to RNT and emotional distress. Impact on questionnaire measures will additionally be assessed at 1-month follow-up. Multigroup analyses will be conducted to assess the impact of CBM-I on near-transfer and far-transfer outcome measures.

**Strengths and limitations of this study**

- One of the first experimental studies to examine two forms of repetitive negative thinking (RNT), specifically worry and rumination, in people with a diagnosis of generalised anxiety disorder or depression, respectively, to investigate the extent to which these different forms of RNT are maintained by common or distinct processes.
- Assessing the causal role of a transdiagnostic cognitive process—interpretation bias—in maintaining different forms of RNT.
- Investigating moderators of multisession home-based cognitive bias modification for interpretations (CBM-I) effectiveness by comparing CBM-I with or without prior activation of RNT.
- Including an active control condition closely matched to training to examine whether modifying negative interpretations is critical for driving effects on interpretation bias, RNT and symptoms of anxiety and depression.
- The brief follow-up period (1 month) limits conclusions regarding long-term impact of training interpretations.

## Background and rationale

Worry and rumination are two forms of repetitive stereotyped thinking characterised by their negative content, overgeneral abstract style and—at pathological levels—by their apparently uncontrollable and perseverative nature. Both are associated with psychological distress, such as heightened anxiety and low mood,^1^ ^2^ and are prevalent in a range of psychological disorders (see ref. 3). Moreover, worry and rumination are central to two particular emotional disorders. Uncontrollable worry about multiple topics is the key diagnostic criterion of generalised anxiety disorder (GAD),^4^ while rumination is a common feature of depression.^5^ Worry and rumination differ in thought content and in aspects such as temporal orientation (eg, future threats in GAD and past/ongoing concerns of failure in depression^6^). Their overlapping characteristics as well as their co-occurrence within individuals and across disorders have led worry and rumination to be conceptualised as a transdiagnostic process^3^ ^7^ termed repetitive negative thinking (RNT). While worry and rumination may be characterised by a common thought process with distinct content, it remains unclear whether they are underpinned by the same cognitive mechanisms. This is especially important to elucidate given the role of RNT in maintaining clinical disorders (see ref. 8) and to inform approaches for therapeutic intervention. The present multisession experimental study aims to investigate interpretation bias—the tendency to make certain interpretations about ambiguous information—as part of a cognitive mechanism underlying RNT in relation to worry in GAD and rumination in depression.

Hirsch and Mathews^9^ identified three processes underlying RNT in the form of pathological worry: namely, emotional processing biases towards negative information, a verbal thinking style and deficits in attentional control. In the current study, we focus on emotional processing biases and specifically on negative interpretation bias, the tendency to habitually interpret ambiguous information as negative or threatening, to investigate whether negative interpretation bias plays a causal role in worry and rumination. Negative interpretations are prevalent in GAD and depression^10–13^ (for a review, see ref. 14). Its causal impact on worry and rumination has been examined using cognitive bias modification for interpretations (CBM-I), an experimental paradigm which repeatedly resolves ambiguous situations (often realistic scenarios capturing situations occurring in daily life) to favour certain (eg, positive) interpretations and thus aims to train a particular interpretive style (see refs. 14–16 for a review). In single experimental CBM-I sessions in clinical participants with GAD and non-clinical participants with high levels of trait worry, selectively training benign interpretations reduces worry,^17^ ^18^ while consistently reinforcing negative interpretations increases state rumination.^19^ These findings point to a causal role of interpretation bias in worry and rumination. Thus, interpretation bias seems a promising candidate process to target using interventions designed to reduce worry and rumination and hence RNT.

Although pivotal for our theoretical understanding, single-session CBM-I studies are unable to address the sustained impact of changing interpretation bias. Multisession CBM-I, providing repeated training over several days to weeks and including a post-training follow-up period, is necessary to investigate the longer term training success of CBM-I on ‘near-transfer’ measures of interpretation bias and ‘far-transfer’ effects relating to central aspects of the disorder such as reductions in RNT, anxiety and depression symptoms. Initial multisession studies training positive interpretations revealed promising effects of CBM-I in reducing anxiety^20–22^ and depression.^23^ ^24^ However, more recent multisession studies have presented mixed findings, with some showing that both CBM-I and active control conditions, in which no training effects are expected (eg, because positive and negative interpretations are presented with equal frequency) reduce negative interpretation bias.^25^ ^26^ It is possible that repeated exposure even to 50% positive interpretations may lead to an incidental training effect over time and to reductions in anxiety and depression symptoms^26^ ^27^ (see ref. 14, online supplementary materials, for considerations regarding the design of CBM-I studies). Thus, active control conditions which are closely matched to aspects of CBM-I (structure of sessions, number of sessions, experimenter contact, etc) but do not target resolution of ambiguity may be needed to demonstrate that effects of CBM-I are indeed driven by changes in interpretations. In the present study, we include an active control condition in which our training materials are adapted to remain ambiguous (no resolution of ambiguity); this was chosen over conditions in which materials are unambiguous (eg, ref. 27, study 2) which have also shown training effects.

Multisession CBM-I has the potential to shed light on the role of interpretation bias in sustaining worry and rumination and was developed to test hypotheses regarding causality. Previous studies on CBM-I have focused on its possible clinical utility (eg, it has been compared to cognitive behavioural therapy^22^ ^26^) with a focus on reducing psychological distress (notably anxiety and depression), rather than with a view of better understanding cognitive mechanisms underlying RNT in clinical disorders. In this vein, although single-session studies have tailored training materials specifically to worry^18^ and rumination-related^19^ concerns, training materials in multisession studies often relate to broader symptoms of anxiety and depression rather than RNT. Thus, it is difficult to draw conclusions about the role of interpretations specifically in relation to RNT. In the present study, we tailor our CBM-I (and control) materials to common worry and rumination concerns with the hope of training interpretations made in the context of RNT (see also ref. 14 regarding the necessity to closely match materials used to assess or modify interpretation bias to ambiguity related to the key constructs—in this case RNT—being investigated).

The importance of considering the context in which interpretations are trained is further highlighted by research into another type of emotional processing bias, namely attentional bias towards negative or threatening information. Engaging in a period of experimentally induced worry in its typical verbal form increases attention to threat cues,^28^ indicating that worry may activate latent cognitive biases. Consistent with this (although in a different disorder), activating concerns in individuals with social anxiety disorder by exposure to idiosyncratic anxiety-provoking situations enhances the effects of subsequent attention training, leading to a greater reduction in social anxiety symptoms.^29^ This may be due to (re-)activation of emotional processing biases, rendering these more malleable and susceptible to modification, akin to findings in the memory reconsolidation literature.^30^

While providing tentative evidence that activating emotional processing biases via RNT (or exposure) may be beneficial in enhancing training effects, this has not yet been explored in relation to CBM-I. This is despite evidence that a depression-specific interpretation bias is most likely to be demonstrated in the context of depressive rumination.^31^ ^32^ Building on this evidence, we seek to examine whether engaging in RNT prior to CBM-I, proposed to activate interpretation bias, will moderate the effects of CBM-I on interpretation bias, RNT and psychological distress. Although the attention bias literature points to a potential beneficial effect of prior RNT, it must be considered that Kuckertz *et al*^29^ focused on social anxiety rather than RNT. RNT, especially in its typical verbal form, depletes attentional control.^33–35^ Thus, it is possible that worrying or ruminating prior to CBM-I may hinder beneficial effects of training. Furthermore, effects of prior RNT may differ for the two diagnostic groups. As the first multisession study to test the proposed moderating role of RNT prior to training, we compare CBM-I with an induced period of RNT before training to CBM-I without such an induction (ie, standard CBM-I). Based on memory reconsolidation studies showing it is necessary to allow 10 min after reactivation for reconsolidation processes to be initiated (see ref. 30), participants in the condition with an induced period of RNT will engage in RNT on a recent RNT topic, followed by CBM-I relating to various worry or rumination topics (see also the Experimental conditions section). To further augment the impact of prior RNT on training, we then present CBM-I materials matched in content to participants' current RNT topic 10 min after their period of RNT. Participants in the standard CBM-I condition will be asked to indicate a recent RNT topic without subsequently engaging in RNT, and will also receive tailored CBM-I materials 10 min after completing a neutral (non-RNT) task.

### Study objectives (rationale and comparators)

This experimental study aims to investigate whether worry and rumination, two forms of RNT, share underlying cognitive mechanisms. We focus on negative interpretation bias, a transdiagnostic cognitive process prevalent in GAD and depression (see ref. 14) and shown to play a causal role in influencing levels of worry^17^ and rumination^19^ in single-session experimental studies. Using CBM-I, we investigate whether repeatedly training positive interpretations over multiple sessions (vs an active control condition) leads to a sustained reduction in worry and rumination in the disorders in which they play central maintaining roles, namely GAD and depression. Individuals with GAD or depression will receive worry-related or rumination-related training materials, respectively, to maximise potential training of interpretations relevant to the key form of RNT for the given disorder. We assess interpretation bias, levels of worry and rumination (primary outcomes) as well as anxiety and depression symptoms (secondary outcomes) at baseline (study visit 1) and again after multisession CBM-I or active control sessions (study visit 2). In addition, we examine the sustained impact of training positive interpretations on levels of worry, rumination, anxiety and depression at 1-month follow-up. Further, we compare two types of CBM-I, with and without prior RNT, to examine whether activating RNT modulates the effects of CBM-I in individuals with GAD and depression. Finally, to consider how cognitive processes may influence each other, and drawing on the combined cognitive bias hypothesis^36^ as well as building on findings that single-session CBM-I increases attentional control during worry,^18^ we include a classic Stroop and emotional Stroop task to examine whether multisession CBM-I impacts the ability to inhibit irrelevant and RNT-related information.

### Hypotheses

For both diagnostic groups, we predict that CBM-I with and without prior RNT will reduce negative interpretation bias, RNT (levels of worry and rumination) and psychological distress (levels of anxiety and depression) relative to the active control condition. Furthermore, we examine whether an induced period of RNT prior to CBM-I either enhances the effects of training (by activating underlying cognitive biases, as in attention bias research), or reduces training effects (potentially due to the additional demands placed on attentional control resources by RNT). Finally, and crucially given its proposed underlying role, we expect that effects of CBM-I on worry and rumination, anxiety and depression will be driven by changes in interpretation bias.

## Methods

### Design

Individuals meeting Diagnostic and Statistical Manual of Mental Disorders Fifth Edition (DSM-V) diagnostic criteria for either GAD or current major depressive episode (MDD) will be randomly allocated to one of three conditions: CBM-I with prior RNT (henceforth CBM_RNT), standard CBM-I without prior RNT (henceforth CBM_STAND) or an active control condition (henceforth CONTROL). To investigate the impact of CBM-I on changing interpretation bias, near transfer will be assessed using measures of interpretation bias. To explore the impact of CBM-I on measures of RNT and psychological distress, far transfer will be assessed by changes to levels of worry and rumination (assessed using a behavioural task and self-report questionnaires) as well as self-report measures of anxiety and depression symptoms following 10 internet-based CBM-I or active control sessions over the course of 3 weeks. Impact on all questionnaire measures will additionally be assessed at 1-month follow-up.

### Participants and recruitment

**Figure 1:**
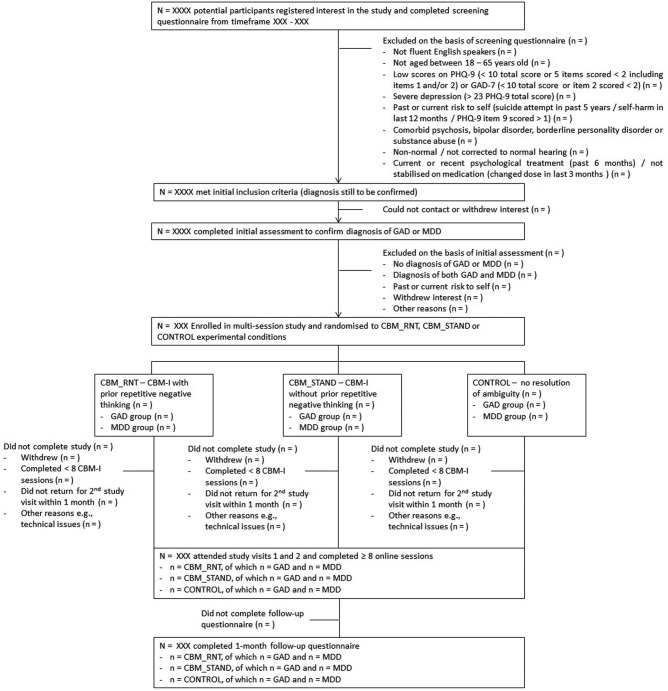
Study flow chart.

Participants with GAD or MDD will be recruited from the community in Greater London via online advertisements and university circular emails. Participants must be able to attend two experimental sessions at the Institute of Psychiatry, Psychology and Neuroscience, King's College London, located in South London (see figure 1 for the study flow chart). Participants will be fluent English speakers aged between 18 and 65 years old. They will be screened for levels of anxiety and/or depression, and participants with a total score <10 or five items scored <2 including items 1 and/or 2 on Patient Health Questionnaire 9 (PHQ-9)^37^ and/or with a total score <10 or item 2 scored <2 on GAD-7^38^ will be excluded. Further exclusion criteria are severe depression (>23 PHQ-9 total score), past or current risk to self (self-harm in past 12 months/suicide attempt in last 5 years/PHQ-9 suicidal ideation item 9 scored >1^39^), comorbid psychosis, bipolar disorder, borderline personality disorder or substance abuse, non-normal/not corrected to normal hearing (as the study involves listening to audio clips) as well as current or recent (past 6 months) psychological treatment. Further, individuals taking psychotropic medication must have been stabilised on that medication for at least 3 months without remission to be included. The latter two criteria serve to ensure that any positive effects of CBM-I are not confounded by ongoing improvements due to other treatments. Diagnosis of GAD or MDD will be confirmed using the Structured Clinical Interview for DSM-V axis I disorders (SCID^40^) at an initial assessment prior to the first experimental session. Authors CK and JW will administer the diagnostic interviews. A clinician will blind-code a subset of interviews to assess reliability. Participants meeting diagnostic criteria for both GAD and MDD, that is, participants with current comorbid GAD and MDD, will be excluded.

#### Sample size

Prior single-session CBM-I research with participants with GAD has indicated that CBM-I training (vs control) had a large effect on the breathing focus thinking task (*f^2^*=0.43,^17^ see below for task description). Using G*POWER software^41^ to calculate sample sizes achieving 80% power at p=0.05, N=49 in each group (16 per condition) would be needed to reveal a large effect size on the breathing focus task. We based our power calculation on this far transfer task because far-transfer is more difficult to achieve than near transfer, and near-transfer effects of multisession CBM-I training have been found to be large (see ref. 42). Based on this calculation, we aim for a final cell size of at least n=20 participants per condition in each diagnostic group.

#### Randomisation and blinding

Participants will be randomised to one of the three conditions on the basis of a random allocation sequence generated on http://www.random.org by a person not involved in the research study. As in Williams, Blackwell, Holmes and Andrews,^39^ numbers 1–3 corresponding to the three conditions will be placed in sealed envelopes, the envelopes marked with the ascending sequence order number, and participants allocated to conditions according to this sequence. Participants will remain blind to the condition until the end of the study (when they will be debriefed). Researchers will open the envelope at the first experimental session once participants have provided written informed consent. Blinding of the researchers will not be possible as researchers guide participants through the first online session, which differs by experimental condition (see below).

### Experimental conditions

All three experimental conditions comprise 10 sessions: 1 initial session at the first study visit and 9 sessions to be completed at home using a purpose-built online platform over the course of 3 weeks (participants are instructed to aim to complete 3 sessions per week). All online sessions begin with the prescenario RNT induction (CBM_RNT) or neutral task (CBM_STAND or CONTROL). Subsequently, participants listen to 50 audio clips (henceforth scenarios) per session and answer a corresponding question after listening to each scenario. All scenarios were audio-recorded by a professional actress and are presented to participants via headphones. In keeping with CBM-I research (see ref. 16), instructions in all conditions emphasise that participants should imagine themselves in the situations the scenarios describe and try to anticipate the ending of each scenario. Mood ratings are obtained after the RNT induction or neutral task and at the end of each session (see also below).

#### Prescenario task: RNT induction and neutral task

*RNT induction:* The RNT induction consists of a 5-min task, adapted from Hertel, Mor, Ferrari, Hunt and Agrawal,^19^ which is completed as part of each online session prior to the CBM-I scenarios in the CBM_RNT condition. Participants first select a theme about which they have found themselves worrying (GAD group) or ruminating (MDD group) on the day of the session or very recently. Participants have the choice of three common themes (performance, social relationships, or illness and harm for GAD worry; abilities, social relationships, or feeling low for depressive rumination) which correspond to the main themes reflected in the CBM-I and control scenarios (see below). Each theme may be selected once a week (three times in total) to ensure that all participants engage in RNT about themes relevant to the training/control materials. Following the selection of the theme, participants rate the frequency with which they have recently (past few days) thought about a topic relating to this theme and how distressing this has been for them. Then, participants are asked to write a one-line summary of their worry/ruminative thoughts on their selected topic, which is fed back to them on a subsequent screen. Participants are next prompted to identify negative thoughts relating to their topic. To facilitate this, participants with GAD see the prompts ‘When you worry about [one-line summary of topic], what do you fear might happen?’, while participants with depression see the prompt ‘When you ruminate about [one-line summary of topic], what negative thoughts do you have?’ Following this, participants are instructed to write down their stream of thoughts for 3 min:

Now, still focusing on **[one-line summary of topic]**, please write down the negative thoughts you usually have when you **worry/ruminate**^[i]^ about this.

Please don't be concerned about grammar or your thoughts jumping around, but rather try and **write down your thoughts about this topic as they naturally occur**. You don't need to provide any background information; just start with the first thought that comes to mind.

We ask you to try and write for the full **three minutes**. After three minutes, the screen will change automatically.

Please begin now.

This 3-min period of writing is intended to help participants activate themes of worry/rumination on the designated topic and is followed by a 2-min period of silent worry/rumination in participants' usual manner (akin to refs. 43 and 44):

Now please continue to think as you usually would about [**one-line summary of topic**] for two minutes.

You can close your eyes to do this, and a beep will sound at the end of the two minutes.

If you notice your mind wandering to thoughts that are not distressing or worrying/ruminative, please try to bring your mind back to worrying/ruminating about [**one-line summary of topic**].

Please press ‘next’ to begin.

Following this, participants complete ratings of their current level of worry, rumination, anxiety and depression, which serve as manipulation checks (see below).

*Neutral task:* To minimise the opportunity for spontaneous RNT and to control for the time that participants are engaged in the RNT task, participants in the CBM_STAND and CONTROL conditions complete a neutral task. This 5-min task consists of reading a short story, divided into eight sections, and making a grammatical correctness judgement about the last sentence of each section. At the end of the eight sections, participants complete a comprehension question about the story content, designed to enhance engagement with the task. The presentation of each section is timed to ensure that the duration of the task is matched to the RNT induction. Participants read a different story at each session (order randomised across participants). Subsequently, participants complete the same ratings of current worry, rumination, anxiety and depression as participants in the CBM_RNT condition. A subset (five) of stories was initially piloted in N=23 participants (unrelated to the current study) and stories were judged to be emotionally neutral (mean valence rating on a 100-point visual analogue scale with anchors *very negative/very positive* =59, SD=1.41).

#### Online scenario-based task: CBM-I and control condition materials

*CBM-I materials*: The two CBM-I conditions (CBM_RNT and CBM_STAND) consist of participants listening to scenarios describing situations relating to common worry-related (GAD group) and rumination-related (MDD group) themes which are initially emotionally ambiguous and which are resolved in either a positive manner (76% of the time), negative manner (12%) or which are left unresolved (12%) by the final word or short phrase of the scenario. Positive scenarios constitute the training scenarios and are designed to promote positive interpretations of ambiguous events; negative scenarios are included to ensure that participants attend carefully to the content of the scenarios. Previous research has shown that negative trials do not interfere with learning and may be helpful by providing an intermittent reinforcement contingency.^16^ ^45^ Following each scenario, participants respond to a Yes/No question related to the content of the scenario. On positive and negative trials, participants receive feedback as to whether their response was correct or incorrect, that is, in line with the intended interpretation provided at the end of each scenario. This serves to reinforce the interpretation. The 12% unresolved scenarios serve as ‘test’ trials. On ‘test’ trials, participants do not receive feedback, but their response is recorded as an indicator of interpretation bias.

Worry scenarios were adapted from Mathews and Mackintosh,^16^ Hirsch, Hayes and Mathews,^18^ Hayes, Hirsch, Krebs and Mathews,^17^ and Elaine Fox (personal communication, 21 July 2015), while rumination scenarios were adapted from Holmes, Mathews, Dalgleish and Mackintosh,^46^ Hertel, Mor, Ferrari, Hunt and Agrawal,^19^ and Blackwell *et al.*^25^ In addition, further scenarios were created by the authors, resulting in 500 unique worry-related and 500 unique rumination-related scenarios. A subset of these scenarios was piloted online in N=92 participants in the general population using the crowdsourcing platform Amazon Mechanical Turk. Scenarios were presented without the disambiguating ending and participants suggested words to complete the scenarios. This approach was taken to ensure that scenarios were (1) well balanced, that is, had the potential to be disambiguated in a positive or negative manner (although during CBM-I, positive interpretations were selectively trained) and (2) were constructed to lead to relatively specific interpretations being made (rather than a wide range of possible interpretations). Scenarios were amended on the basis of these responses and a further subset was reviewed by expert researchers in rumination and worry as well as individuals with lived experience of GAD and depression.

All scenarios are around 50 words long. Worry-related scenarios cover three main themes of worry, namely performance, social relationships and physical threat, including illness and harm, as well as other common themes such as financial worries.^47^ ^48^ Rumination-related scenarios cover three main rumination themes, namely feeling low (ie, ruminating about depressive symptoms^49^), abilities (relating to personal failure^5^) and social relationships (eg, ref. 50) as well as other themes linked to depressive symptoms such as feelings of guilt/shame. Example worry and rumination-related scenarios with corresponding questions are presented in table 1.

**Table 1:** Example scenarios for worry-related and rumination-related CBM-I and active control conditions

	Scenario type	Scenario	Benign ending*	Negative ending†	Question	Benign/correct answer
Worry	Social relationships	You haven’t socialised much with the other members of your fitness class but following a good workout, you all go out for lunch. Later, when you are walking out of the restaurant, you overhear two members of the class talking about you. As you listen, you hear them saying that they think you are…	Alright	Boring	Do the other team members dislike you?	No
	Performance	You are learning how to use a new piece of software and have been completing tutorials online. The test is coming up in a few days and thinking about the amount of effort you have put in, you know you will do…	Very well	Badly	Will you fail the test?	No
	Illness and harm	You are walking down the road to your house late at night when you fall over and scrape your knee. When you look up, a group of teenagers are walking towards you. One of them reaches into their pocket and pulls out a…	Tissue	Knife	Did the teenagers help you?	Yes
	Test‡	Your flatmate is working late so you are in the house on your own this evening. You have just switched off the lights and got into bed when you hear a scraping in the lock of the front door. As you slowly approach the front door, it opens and you see what was causing the noise.	–	–	Is your flatmate home from work?	Yes
Rumination	Social relationships	At the bus stop, you bump into someone from your school days. They comment on how much you have changed since they last saw you. Mulling it over later, you think about their tone of voice and decide that they meant their comment to be…	Complimentary	Hurtful	Did the person intend to be mean?	No
	Abilities	You overhear someone at work saying that the company is making a few people redundant. You start thinking about the contribution you have made to the team since you started working at the company, and as you think it over, you suspect that you will be…	Safe	Made redundant	Will you be sacked?	No
	Feeling low	You are feeling quite exhausted. On social media, you see a photo of you when you felt better. As you think back to that time, you wonder whether in the future there will be times when you will feel less exhausted and you can see that there is…	Hope	Little prospect	Will you always be tired?	No
	Test	Your work contract is coming to an end. A colleague offers to organise leaving drinks for you and they email round the office to ask who is free. As you think back over your relationships with your colleagues, you can guess how many people are likely to come along.	–	–	Will a lot of people come along to your leaving do?	Yes
Control§	Ambiguous	You went on holiday with two friends. Your friends planned to go to a particular place, but you'd been there before and so a new destination was found. Looking back, you wonder whether your friends minded changing the plans for you.	–	–	Did your friends resent having to change the plans?	No
	Fact	Your mother lends you her brand new suitcase to take on a plane journey. You try to look after it but when you arrive at your destination, you see the case is damaged. As you contemplate telling your mother, you know who she will blame for the damage.	–	–	Did you take the suitcase on the train?	No

*In 76% of trials, the scenarios are provided with a benign ending.

†In 12% of trials, the scenarios are provided with a negative ending.

‡Test trials remain ambiguous and make up the final 12% of trials. Participants’ response to the test trial question indicates the interpretation they have made.

§While all CONTROL scenarios remain ambiguous, 50% are followed by questions that assess participants’ interpretation of the scenario (including disorder-specific test trials as above) and 50% relate to other fact-based information in the scenario that is not ambiguous and is emotionally neutral.

Within each online session, scenarios are presented in a pseudorandom order. The first half of each session (first 25 scenarios) includes scenarios from all themes. The second half of each session (remaining 25 scenarios) is matched to participants' concerns on the day. Participants select one of the main themes outlined above at the start of the session; this is also the theme participants will worry/ruminate about if they are in the CBM_RNT condition. The second half of the session then primarily presents scenarios relating to the selected theme. The number of different trial types (positive/negative/‘test’ trials) is kept constant in both halves of the session.

*Active control materials:* To control for listening to scenarios and answering questions relating to the scenarios, the control condition consists of participants hearing ambiguous scenarios in which the ambiguity is not resolved, and which thus should not change interpretation bias. To match the control condition as closely as possible to the CBM-I conditions, each session contains scenarios adapted from worry and rumination materials, with the endings amended so that the ambiguity is left unresolved. As in the CBM-I conditions, participants hear 50 scenarios per session. Each scenario is either followed by a Yes/No question relating to the ambiguity of the scenario, which is not followed by feedback (similar to the ‘test’ trials), or relating to a factual element of the scenario, which is followed by feedback regarding whether participants' responses were incorrect. Both questions are designed to encourage participants to process the meaning of the scenarios. In addition, the ‘test’ trials from the CBM-I conditions will be included so that interpretations made across the course of the sessions can be compared between CBM-I and control conditions. Thus, we can also examine whether participants in the control condition impose their (default) negative interpretive style on the scenarios and whether there are any inadvertent training effects, such as participants in the control condition generating more positive or negative interpretations across the sessions. Example control scenarios with the different question types are presented in table 1.

### Primary outcome measures

#### Interpretation bias (near transfer)

*Scrambled sentences test* (based on ref. 51): The scrambled sentences test involves reordering a list of words to form sentences. Participants use five of six presented words to form grammatically correct sentences, which can either be of negative or positive valence. For example, ‘looks the future bright very dismal’ may be unscrambled to form the sentence ‘the future looks very bright’ (positive) or ‘the future looks very dismal’ (negative). Participants are told to form the first sentence that comes to mind (rather than select a particular valence). In the present study, participants unscramble 20 sentences in 5 min, while holding in mind a string of six digits, as such a cognitive load depletes cognitive resources and is thus thought to present a truer reflection of participants' interpretative style.^51^ Half the sentences are related to worry themes (see themes above) and were generated by the authors, while half are related to depressive rumination and were selected from Wenzlaff and Bates^51^ and Wenzlaff and Bates.^52^ The number of negative sentences divided by the total number of grammatically correct sentences generated serves as an index of interpretation bias, with a higher index (scores range from 0 to 1) denoting a more negative interpretation bias. The test can be scored across all items or for worry and depressive rumination items separately. The scrambled sentences test is administered at the first and second study visit; thus, two separate lists of 20 items were created and the order is counterbalanced across participants.

*Recognition memory test* (based on ref. 16): The recognition memory test consists of two parts. In the first part, participants read 20 ambiguous scenarios, make a fragment completion judgement on the final word (see example below) and answer a comprehension question for each scenario. In the second part, participants are prompted with the title of each scenario and are presented with four statements for each scenario. Two statements relate to the resolution of the ambiguity in the scenario (targets), while the other two statements do not relate to the ambiguity (foils). In each pair of targets and foils, one statement is positive and one negative in valence. Participants rate how similar each presented statement is to the content of the original scenario, with greater similarity ratings for positive targets indicating a more positive interpretation of that scenario (and vice versa for negative targets). Foils are included to assess a general valence effect. The recognition test is relatively opaque in terms of its purpose and is not subject to demand or selection bias (see ref. 14 online supplementary materials A).

Of the 20 scenarios, half are related to worry (taken from refs. 16 and 46, with some slight wording changes) and half relate to depressive rumination (created by the authors). An example item developed for the experiment is shown below, followed by the corresponding target and foil statements (A–D).

## The day trip

Friends of yours have organised a day trip. You have been feeling quite low and wonder whether to join them. In the end, you decide to go along. Afterwards, you think back over the day and how going along made you (f e – l) [feel].

Did you go on a day trip with your family? (Yes/No)

- You felt better after going on the day trip (positive target)
- Going on the day trip made you feel worse (negative target)
- The car journey went very smoothly (positive foil)
- The car broke down on the way home (negative foil)

Participants complete the recognition memory test at the beginning and end of the first study visit (before and after the first online session) and at the second study visit; hence, three separate lists of 20 items were generated and the order is counterbalanced across participants.

### 

#### Levels of worry and rumination (far transfer)

*Breathing focus task*: The breathing focus task measures the frequency of negative thought intrusions, which often initiate RNT. Based on the original breathing focus task/worry task,^17^ ^18^ ^53^ participants focus on their breathing for 5 min and indicate at randomly cued intervals whether they are indeed focusing on their breathing or are experiencing a thought intrusion. Participants categorise thought intrusions as negative or otherwise and provide brief summaries of content. Participants then engage in worry (GAD group) or rumination (MDD group) about a salient current worry/rumination topic for 5 min, followed by another 5 min breathing focus period, with sampling as before. After each breathing focus period, participants are reminded of their summaries of each thought intrusion in turn and are asked to provide further details on their thoughts as they originally occurred. These expanded descriptions are audio-recorded and will later be categorised as negative or otherwise by an assessor who is blind to diagnostic group, condition and breathing phase (preperiod vs postperiod of worry/rumination).

*Self-report questionnaires*: Trait worry levels will be assessed using the Penn State Worry Questionnaire (PSWQ^54^) which comprises 16 items (eg, ‘If I do not have enough time to do everything, I do not worry about it’) rated on a scale from 1 (*not at all typical of me*) to 5 (*very typical of me*) and summed (after reverse-scoring appropriate items) to produce an overall score, with higher scores denoting greater worry. Trait rumination will be measured using the Ruminative Response Scale (RRS^55^), which comprises 22 items relating to the frequency of ruminative thoughts (eg, ‘think about how you don't seem to feel anything anymore’) rated on a scale from 1 (*almost never*) to 4 (*almost always*) and summed, with higher scores denoting greater rumination. Participants will also complete a 15-item ‘RNT questionnaire’ (RNTQ) developed for the study that assesses key aspects of RNT and its consequences (eg, frequency of RNT; extent to which participants actively try not to engage in RNT; degree of difficulty controlling RNT; ability to concentrate; extent of catastrophising). Items will be completed in relation to the previous week.

### Secondary outcomes

#### Levels of anxiety and depression (far transfer)

Depressive symptoms will be assessed with PHQ-9^37^ and anxiety symptoms will be assessed using the GAD-7.^38^ These measures are widely used in research and clinical settings, allowing us to draw comparisons with experimental studies and clinical interventions.

#### Inhibition of distracting emotional information

*Classic Stroop and emotional Stroop tasks:* The classic Stroop task^56^ consists of presenting a list of colour words printed in different coloured ink with the instruction to read aloud the ink colour rather than the word itself. The first list (congruent) shows colour words printed in their congruent ink colour (eg, ‘blue’ written in blue ink); the second list (incongruent) features colour words printed in an incongruent ink colour (eg, ‘blue’ written in green ink) and participants must inhibit the meaning of the word to succeed in reading the ink colour. The ink colours and words used in the present study are ‘red’, ‘white’, ‘yellow’, ‘green’ and ‘blue’. Words are presented on a black background. The classic Stroop is followed by an emotional Stroop task, in which three lists, namely, anxiety-related (eg, nervous, criticised), depression-related (eg, tearful, despair) or neutral (eg, umbrella, writing) words are presented in varying ink colours. We drew on previous studies^57–60^ to construct the emotional Stroop; each word list comprised 15 words. Lists were matched for word length and frequency in the English language. Words were repeated five times and printed in five colours (same colours as for classic Stroop above); thus, each list comprised 75 words printed in a pseudorandom order in five vertical columns of 15 words each against a black background. The order of presentation for the different word lists is counterbalanced across participants and study visits. For classic and emotional Stroop tasks, participants read the ink colour out loud to the experimenter. The number of errors made and time latencies (seconds) are recorded for each list; time latencies constitute the outcome variable, and classic and emotional Stroop tasks are analysed separately.

### Additional measures relating to the experimental conditions

*Expectancy and acceptability ratings:* Items to measure study expectancy and acceptability will be adapted from Williams, Blackwell, Holmes and Andrews.^39^ At the first study visit, participants will be asked, ‘At this point, how logical does the programme offered to you seem?’ and ‘How useful do you think this programme will be in reducing your depression/anxiety symptoms?’; at the second study visit, they will be asked, ‘After having completed the programme, how logical was the programme offered to you?’ and ‘How useful was this programme in reducing your depression/anxiety symptoms?’. Participants respond on a five-point scale from 0 (*not at all logical/useful*) to 4 (*very logical/useful*).

*Assimilation and imagination ratings*: Participants will complete nine items adapted from Standage, Harris and Fox^61^ and Holmes, Mathews, Dalgleish and Mackintosh^46^ to investigate the degree to which they assimilate the content of scenarios (‘While listening to the scenarios, how much did you think they were describing reactions that you could not imagine yourself having?’), are able to imagine themselves in the scenarios (‘While listening to the scenarios, to what extent could you see yourself in the scenarios, as though they were happening to you?’) and how vividly they were able to imagine the scenarios (‘How vividly did you imagine the situations that were described?’) on visual analogue scales (0–100).

*Ratings of worry, rumination, anxiety and depression during CBM-I/control sessions (manipulation checks)*: During each online session, participants will complete ratings of worry, rumination, anxiety and depression immediately after the worry/rumination induction/filler task, and at the end of the session. Single-item questions assessing current level of worry, rumination, anxiety and depression will be presented as visual analogue scales (0–100). Mean ratings will be compared across conditions to assess that the worry/rumination induction differs from the neutral task as intended.

### Procedure

**Figure 2:**
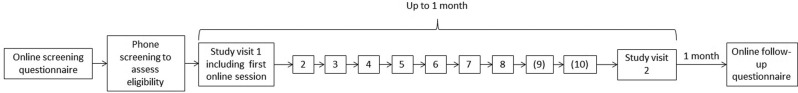
Participant flow chart with measurement points.

Participants will be invited to take part in a research study into worry and rumination which includes two face-two-face study visits scheduled 3 weeks apart, nine homework sessions to be completed online between study visits and a follow-up questionnaire to be completed online 1 month after the second study visit (see figure 2 for participant flow through the components of the study). Prospective participants will be recruited from the community and from King's College London. They will complete an initial screening questionnaire online, including the PSWQ, RRS, PHQ-9 and GAD-7. If they meet inclusion criteria, the SCID will be completed by a trained researcher to confirm a diagnosis of GAD or depression. Once enrolled, participants will complete a presession questionnaire including standardised measures (PSWQ, RRS, PHQ-9, GAD-7, RNTQ) as well as demographic information online within 24 hours prior to the first study visit (experimental session). Should participants indicate risk to themselves (a score of >1 on PHQ-9 suicidal ideation item) on the preassessment questionnaire, this will trigger a risk assessment at the study visit.

At the first study visit (experimental session), participants provide written informed consent and will be randomised by diagnostic group to one of the three conditions: (1) CBM_RNT, (2) CBM_STAND or (3) CONTROL (see the Experimental conditions section above for details). They will then complete the scrambled sentences test, classic and emotional Stroop task, recognition memory test and breathing focus task. Then, participants will be given the study rationale for the online homework sessions and will complete expectancy and acceptability ratings before completing the first online session on the study website. This is completed during the study visit to ensure participants understand the instructions for their condition and are able to complete the sessions correctly. Each session includes the RNT induction (CBM_RNT) or neutral task (CBM_STAND and CONTROL conditions), followed by 50 scenario audio clips (see above) with a short break after 25 scenarios. In addition, participants complete ratings of state levels of worry, rumination, anxiety and depression after the prescenario task (RNT or neutral task) and at the end of each session. Following the first online session, participants complete the recognition memory test again (different scenarios; see above) to assess change from pre to post first study visit. Finally, participants are presented with instructions for completing the nine online sessions over the course of the next 3 weeks, and the researcher will complete a diary card with each participant to help schedule the homework sessions.

Participants will complete three online sessions per week over the next 3 weeks, with some flexibility in terms of dates and times of day, and with the provisos that they complete no more than one homework session per day and complete the homework sessions within 1 month and the final session no later than the day before their second study visit. Researchers will monitor adherence to the homework sessions using the online platform. They will keep in touch with participants by using participants' preferred method of contact (email, phone, SMS) to facilitate engagement and trouble shoot issues with the online sessions, and will encourage participants to catch up missed sessions. Participants are required to complete a minimum of eight sessions (see also the Statistical methods section).

Up to 24 hours before returning for their second study visit, participants will again complete the presession questionnaire (PSWQ, RRS, PHQ-9, GAD-7, RNTQ), and additionally will complete a ‘negative events form’ in relation to the previous 3 weeks to assess incidence of mental health-related events (eg, deterioration in symptoms, suicidal intent, hospitalisation, etc). Any events reported will be addressed with the participant at the second study visit; events may also be captured during other points of the study and will be addressed with the participant when they are raised. If the event is related to an aspect of the study, an adverse events form will be generated, which will be reviewed by the Research Steering Committee. At the second study visit, participants will again provide written informed consent for this visit, will complete treatment expectancy and acceptability ratings, assimilation and imagination ratings (in reference to the past nine sessions), and will then complete the scrambled sentences test, classic and emotional Stroop task, recognition memory test and breathing focus task before being debriefed and partially paid for their participation to date. One month after their second study visit, participants will complete the follow-up questionnaire online, which is identical to the presession questionnaire for study visit 2, and receive the final payment for their participation. The experimenter will contact participants for further information should any negative events and/or mental health-related events be reported at follow-up. Participants will receive £130 for their participation in the study.

### Monitoring adverse events

Adverse events (significant and prolonged increases in anxiety and/or depressive symptoms related to taking part in the study) will be recorded and reported to the Research Steering Committee at the end of the study. Serious adverse events (potentially life-threatening events, eg, suicide attempt, hospitalisation due to mental health, death, related to taking part in the study) will be reported immediately to the Chair and Deputy Chair of the Research Steering Committee. In addition, we will record other negative life events reported by participants.

### Data entry, coding, security and storage

The research will be conducted in compliance with data management and sharing policies set out by the Institute of Psychiatry, Psychology and Neuroscience, King's College London, and Nature Life Sciences reproducibility guidelines. Clinical data (eg, questionnaires; SCID forms) will be managed according to Mental Health Research Network (MHRN) guidance. All research data will be coded and anonymised and stored securely in accordance with the Data Protection Act 1998.

### Statistical methods

Statistical analyses will be carried out in Stata V.14 (StataCorp. Stata Statistical Software: Release 14. 2015, College Station, Texas, USA: StataCorp LP) and MPlus.^62^ All variables will be described by diagnostic group, experimental condition and assessment time point. In addition, assessments of feasibility and acceptability in terms of recruitment, drop-out and rate of completion will be summarised, and additional measures relating to the experimental materials will be evaluated to check the manipulations worked as intended.

As this experimental study is designed to test hypotheses regarding the causal role of interpretation bias in worry and rumination in GAD and depression, analyses will be conducted using data from ‘completers’ only, defined as participants who complete ≥8/10 sessions (percentage judged to be an adequate ‘dose’ in previous multisession CBM-I studies^25^ ^26^) and who return for the second study visit within 1 month of their first visit.

To consider differences between GAD and MDD diagnostic groups that received tailored training materials, we will specify multigroup models with diagnostic group as grouping variable. We will test whether parameters differ significantly between the two diagnostic groups. Further, we can examine significant effects within each diagnostic group. Predictors in each model are experimental condition (CBM_RNT, CBM_STAND, CONTROL) and time point (study visits 1, 2 and follow-up; follow-up for questionnaire measures only).

We will examine effects on measures of interpretation bias to assess whether the CBM-I conditions are successful in training positive interpretations (near transfer). We will then assess effects on measures of RNT (self-report and behavioural) as well as anxiety and depression to investigate the impact of our CBM-I conditions on changing RNT, anxiety and depressive symptoms (far transfer). Further, we will examine whether the extent of change on self-report measures of anxiety and depression falls within the range of reliable change, defined as a reduction in scores exceeding the reliable change index for that measure. We will supplement the above analyses with disorder-specific analyses, examining the effects on near transfer for disorder-specific items only (see the Interpretation bias measures). We will control for differences in demographic variables as appropriate by including them as covariates in analyses.

To examine the mediating role of interpretation bias, we will conduct mediation analyses using Baron and Kenny's^63^ approach. Depending on the final sample size and distribution of the data, bootstrapped SEs and bootstrapped CIs will be estimated.

## Discussion

This experimental study investigates interpretation bias as part of a transdiagnostic cognitive mechanism underlying worry and rumination. Using multisession CBM-I tailored towards worry-related and rumination-related concerns, we examine whether repeatedly training positive interpretations (vs an active control condition) reduces negative interpretation bias and leads to a sustained reduction (up to 1-month follow-up) in worry and rumination in the disorders in which they play a central maintaining role, namely GAD and depression. To the best of our knowledge, this is the first study to examine CBM-I in relation to worry and rumination within the same study, allowing us to directly compare the effects of interpretation training on these forms of RNT and elucidate whether they share common underlying processes.

Furthermore, we compare two versions of CBM-I with and without RNT prior to training to investigate whether initiating RNT—proposed to activate latent negative interpretation bias—enhances the effects of CBM-I (as has been shown in cognitive bias modification for attention^29^) or decreases its effects, potentially by depleting participants' attentional control resources (see ref. 35). A further possibility is that the two CBM-I conditions may be differentially effective in GAD compared with depression, that is, that prior RNT is beneficial for one disorder but detrimental for the other. Assessing contextual effects of prior RNT is especially important given the need to better understand moderators and mediators of CBM-I^14^ in order to optimise interpretation training. This can ultimately enrich our understanding of the cognitive processes underlying RNT in emotional disorders such as GAD and depression, and improve CBM-I approaches as potential adjunct interventions alongside more established clinical treatments.

## Ethics and dissemination

The results will be disseminated through conference presentations and publication in peer-reviewed journals. Access to raw data and participant information will be available only to members of the research team.

## Protocol version and status

This is protocol V.1. The experiment is currently recruiting participants. Data collection started in late January 2016. On the date the article was submitted, N=59 participants had completed the study. We anticipate that data collection will be completed in January 2017.

## Footnotes

i Depending on diagnostic group.
